# Evaluation of the effects of humic acids on maize root architecture by label-free proteomics analysis

**DOI:** 10.1038/s41598-019-48509-2

**Published:** 2019-08-19

**Authors:** Rosane Oliveira Nunes, Giselli Abrahão Domiciano, Wilber Sousa Alves, Ana Claudia Amaral Melo, Fábio Cesar Sousa Nogueira, Luciano Pasqualoto Canellas, Fábio Lopes Olivares, Russolina Benedeta Zingali, Márcia Regina Soares

**Affiliations:** 1 Department of Biochemistry, Institute of Chemistry, Federal University of Rio de Janeiro, Rio de Janeiro, Brazil; 2 Biological Inputs to Agriculture Development Center, State University of Northern of Rio de Janeiro, UENF, Rio de Janeiro, Brazil; 3 Medical Biochemistry Institute Leopoldo De Meis, Federal University of Rio de Janeiro, Rio de Janeiro, Brazil

**Keywords:** Plant biotechnology, Proteomics

## Abstract

Humic substances have been widely used as plant growth promoters to improve the yield of agricultural crops. However, the mechanisms underlying this effect remain unclear. Root soluble protein profiles in plants 11 days after planting and cultivated with and without humic acids (HA, 50 mg CL^−1^), were analyzed using the label-free quantitative proteomic approach. Cultivation of maize with HA resulted in higher fresh weight of roots than in untreated plants (control). Plants treated with HA showed increased number, diameter and length of roots. In the proteomics analysis, differences were detected in the following categories: energy metabolism, cytoskeleton, cellular transport, conformation and degradation of proteins, and DNA replication. Thirty-four proteins were significantly more abundant in the seedlings treated with HA, whereas only nine proteins were abundant in the control. The effects on root architecture, such as the induction of lateral roots and biomass increase were accompanied by changes in the energy metabolism-associated proteins. The results show that the main effect of HA is protective, mainly associated with increased expression of the 2-cys peroxidase, putative VHS/GAT, and glutathione proteins. Indeed, these proteins had the highest fold-difference. Overall, these results improve our understanding of the molecular mechanisms of HA-promoted plant growth.

## Introduction

Humic substances (HS) are a major component of soil organic matter, acting as an essential link between various chemical, physical, and biological properties of soil. These properties contribute towards the transportation, mobilization, and dissolution of metals and organics in the environment^1^. When applied directly to plants, soluble HS can exert various morphological, physiological and biochemical effects^2^; thus, HS are increasingly being used as bio-stimulants or plant growth promoters^3^. However, the biological activity of HS and the molecular mechanisms through which they affect plant metabolism remain unclear.

HS can be characterized as relatively small organic molecules that are loosely bound by intermolecular hydrophobic interactions^4^ that are easily disrupted by organic acids. Some components of HS in the rhizosphere can access plant cell receptors, modifying their metabolism. The mechanisms by which HS affect plant metabolism are not yet clear; however, their putative involvement in the regulation of protein synthesis is one possibility. Humic acids (HA) are the components of HS that are formed by associations of predominantly hydrophobic compounds (polymethylene chains, fatty acids, phenolic and steroid compounds) that are stabilized at neutral pH by hydrophobic dispersive forces (van der Waals, π-π bonding, and CH–π bonding)^4^. The conformations of these compounds increase in size as intermolecular hydrogen bonds form at lower pH values, until the point of flocculation^4^.

Traditionally, the effects of HS on plant nutrition have been attributed to metal ion complexes that enhance the availability of micronutrients with low solubility, such as iron and zinc^5^. Moreover, HS have been observed to induce the synthesis of plasma membrane (PM) H^+^-ATPase^6^. This enzyme couples ATP hydrolysis with H^+^ transport across the cell membrane, which acidifies the apoplasts, loosens the cell walls and elongates cells^7^, thereby increasing root growth. The activation of PM H^+^-ATPase also improves plant nutrition by increasing the electrochemical proton gradient that drives ion transport across cell membranes^8^. Tomato roots treated with HS overexpressed high affinity phosphate transporters^9^, which are usually induced transcriptionally by phosphate starvation. However, in the presence of HS, phosphate transporters were induced independently of phosphate concentration, showing unusual changes in protein synthesis programming in response to HS.

Proteomic analysis is a tool for identifying proteins and can be used to evaluate the status of growth and development, cell division and differentiation, and cell responses to abiotic and biotic stress^10,11^. Thus far, there has been only one study of the plasma membrane subproteome of root maize treated with HS^12^, and it shows that the majority of proteins are downregulated in response to HS. In our study, a label-free quantitative proteomic approach was used to understand the molecular mechanisms underlying the effects of HA on total protein expression in the root. This methodology is a large-scale strategy for protein identification in complex mixtures that involves the pre-digestion of intact proteins followed by peptide separation, fragmentation in a mass spectrometer and database search.

We investigated the total root proteome of *Zea mays* (DKB789) seedlings, harvested 11 days after planting, with or without HA. The results showed that the application of HA positively affects the expression of stress-related proteins of plants.

## Results

### Fresh weight of the roots of maize seedlings grown under two conditions

**Figure 1:**
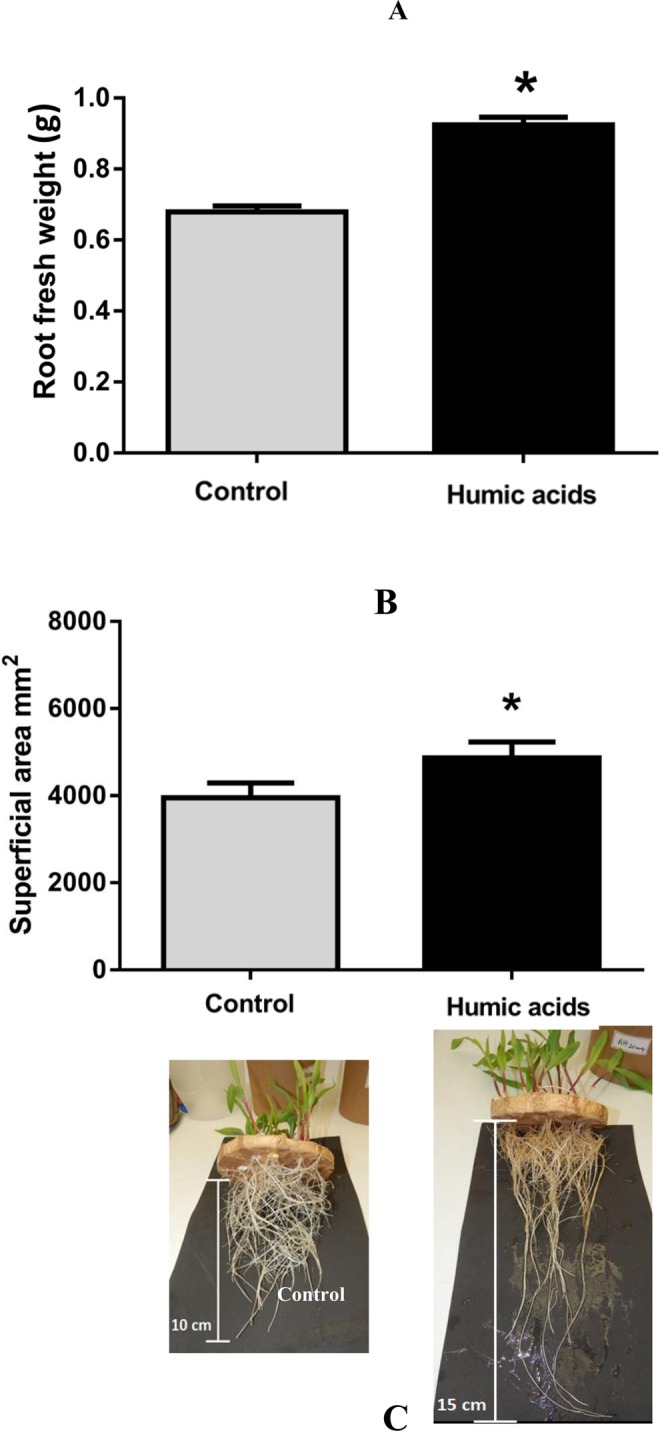
Fresh weight of the roots of maize seedlings grown under two conditions. Each column represents the mean and standard deviation of the thirty plants (**A**). Radicular superficial area of maize seedlings analyzed by SAFIRA software. Each column represents the mean and standard deviation of fifteen plants (**B**). Statistical analysis of the three replicates using the t-test. Asterisks indicate significant differences at p < 0.05. In the photos in (**C**), it is possible to see the difference in maize roots.

The addition of HA to the culture medium increased both the fresh weight and superficial area of maize roots compared with the control (Fig. 1). As shown in Fig. 1A, the root fresh weight of maize seedlings treated with HA was higher than in the plants treated without the addition of HA. In Fig. 1B, the data show that the surface area of the maize roots treated with HA also increased significantly (p < 0.05) in relation to the control plants. Thus, the use of HA promoted growth in maize seedlings (Fig. 1C).

### Protein identification and functional categorization

**Figure 2:**
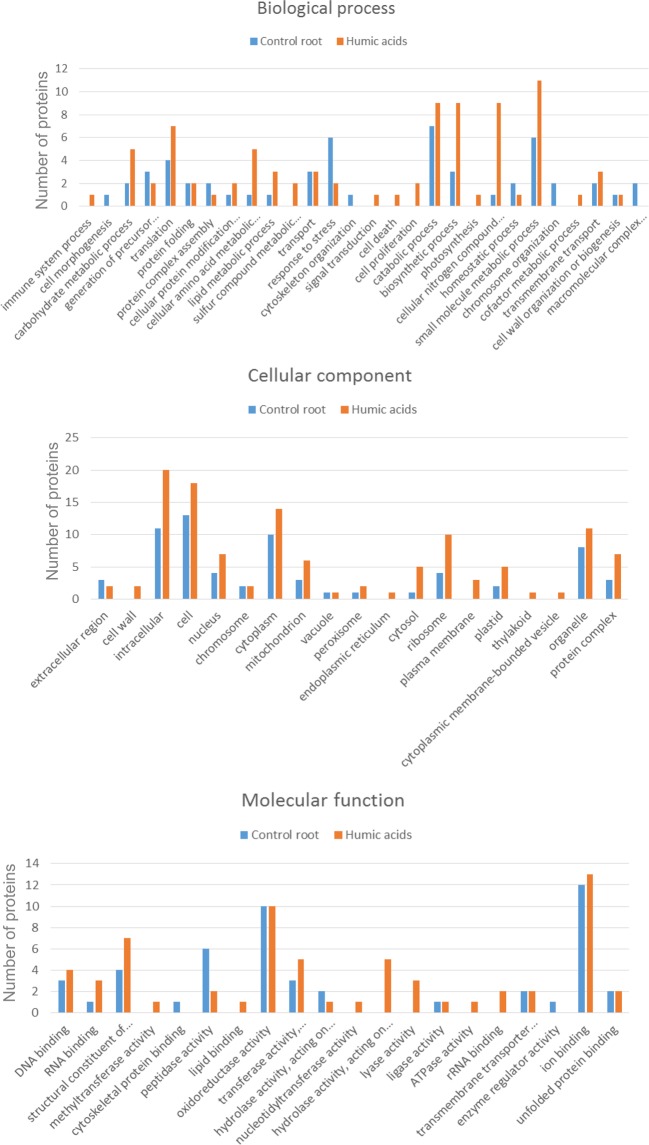
Plant GO Slim was used to summarize the subcategories of the identified proteins. Gene ontology (GO) categories of the proteins detected in maize treated with HA and control plants. (**A**) GO-Molecular function. (**B**) GO-Cellular component. (**C**) GO-Biological process.

A total of 465 proteins were identified (Supplementary Table 1). Gene Ontology analysis identified 67 functional subcategories of proteins (http://www.agbase.msstate.edu) and three primary categories: cellular components, molecular function, and biological processes (Fig. 2). Proteins in some categories were found only in maize roots treated with HA, including those involved in cell proliferation and signal transduction. In the molecular function category, plants treated with HA expressed proteins associated with hydrolase activity, lyase activity, ATPase activity, rRNA binding, RNA binding and the structure of ribosomes. In the cellular component category, plants treated with HA had more intracellular proteins associated with the nucleus, cytoplasm, ribosomes, and other organelles than control plants. Furthermore, cellular component proteins associated with the cell wall, plasma membrane, endoplasmic reticulum, thylakoid membrane and cytoplasmic membrane-bound vesicles were identified only in plants cultivated with HA.

### GO enrichment and pathway analysis of exclusive proteins

**Figure 3:**
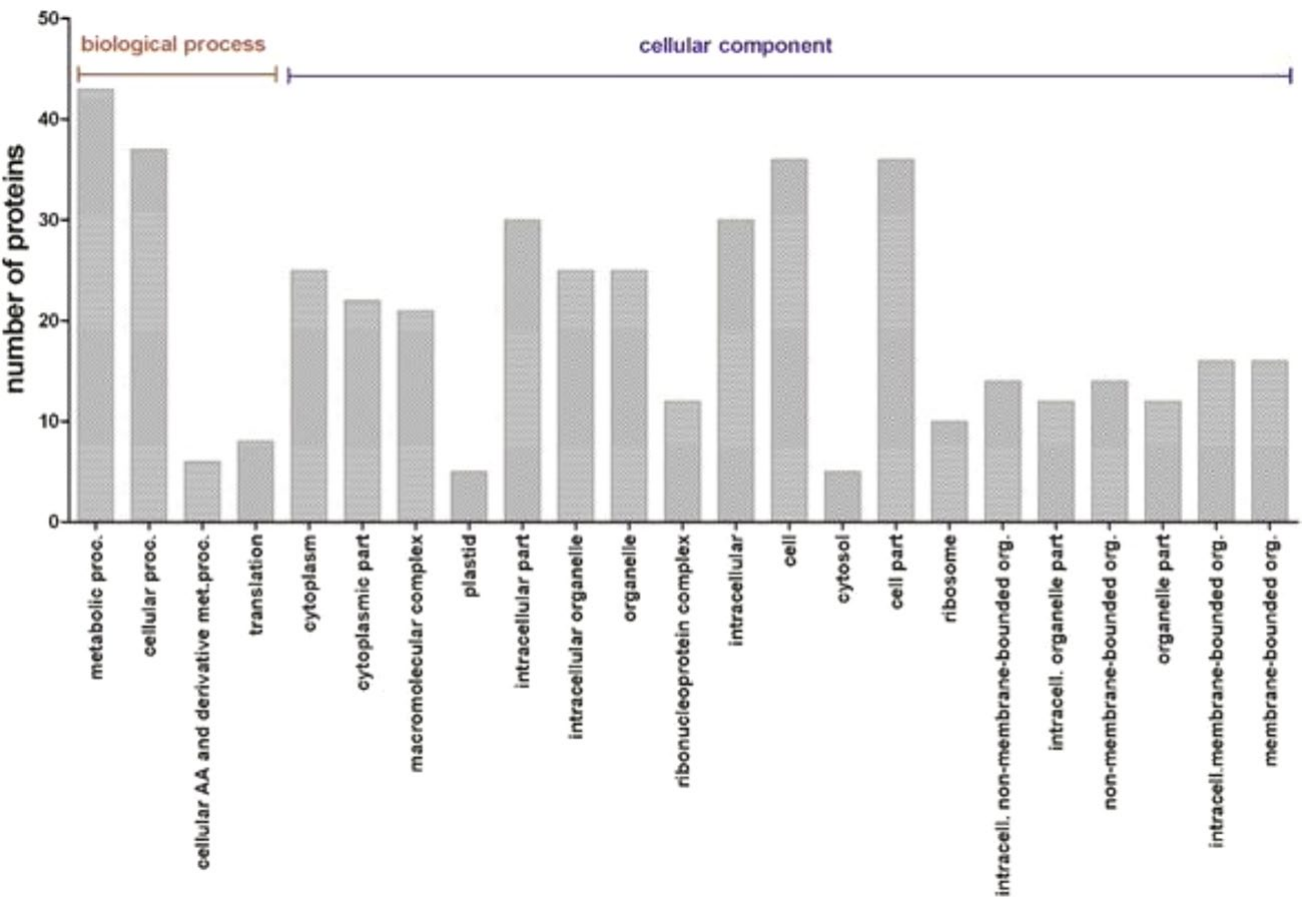
Functional enrichment analysis was used to determine the biological relevance of root proteins using the Singular Enrichment Analysis tool AgriGo. Maize roots cultivated with HA were enriched for GO traits associated with the metabolic processes, ribosome, intracellular components, endoplasmic reticulum, plasma membrane and cytoplasmic membrane-bounded vesicles.

Fifty-six proteins were detected exclusively in roots from maize plants cultivated with HA. Functional enrichment analyses were used to determine the biological relevance of these proteins using the Singular Enrichment Analysis tool AgriGO^13^. Among the biological process, the expressed proteins were strongly enriched for GO traits associated with metabolic processes (GO: 0008152, false discovery range (FDR = 5.1e-05). In the cellular component category, maize roots cultivated with HA were enriched for traits associated with the ribosome, intracellular components, endoplasmic reticulum, plasma membrane and cytoplasmic membrane-bounded vesicles (Fig. 3).

### Quantitative proteomic analysis

**Figure 4:**
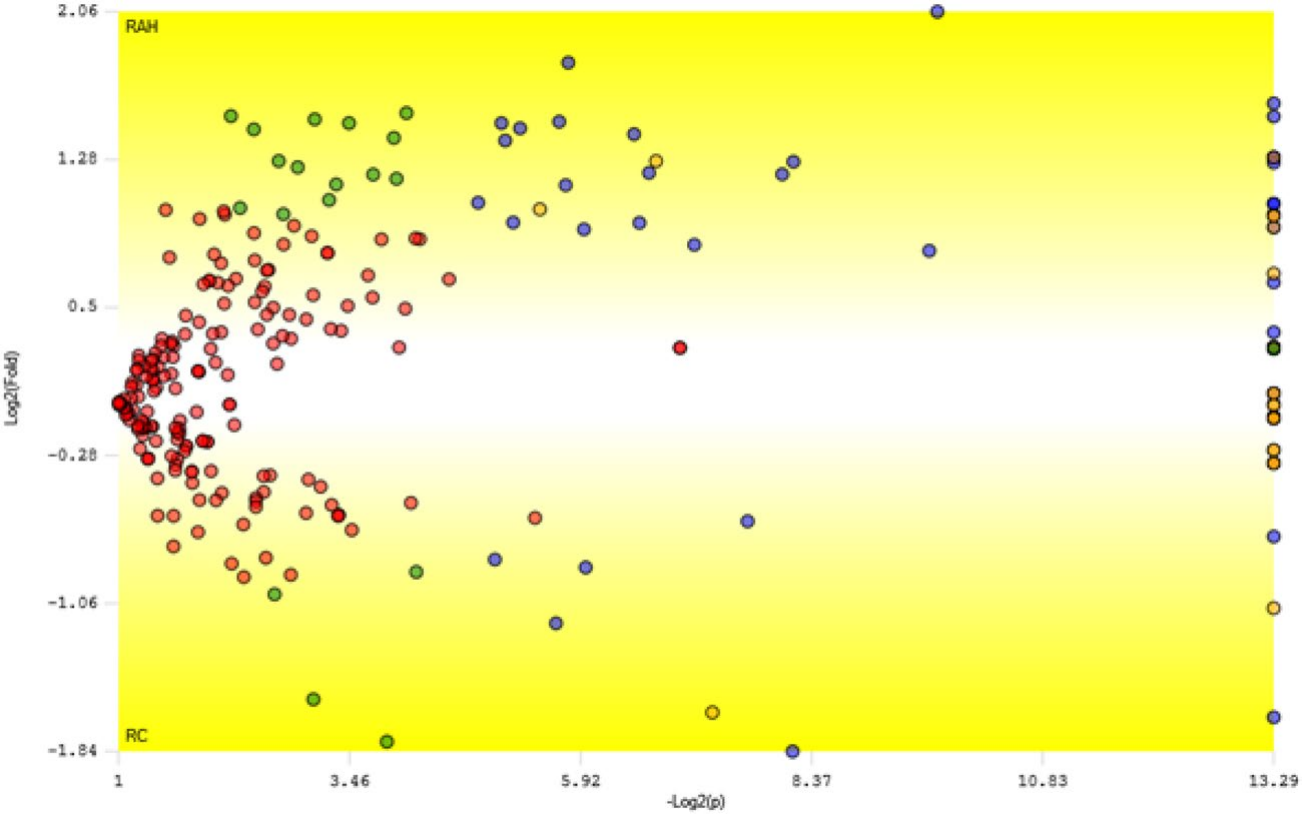
Volcano plot of differentially expressed proteins generated by the PatternLab program. The Y-axis shows the log2 of fold-change of the quotient HAR/CR. The X-axis shows the −log10 of the calculated probability (p-value). Blue dots represent differentially expressed proteins that satisfied both the automatic fold and statistical criteria.

**Figure 5:**
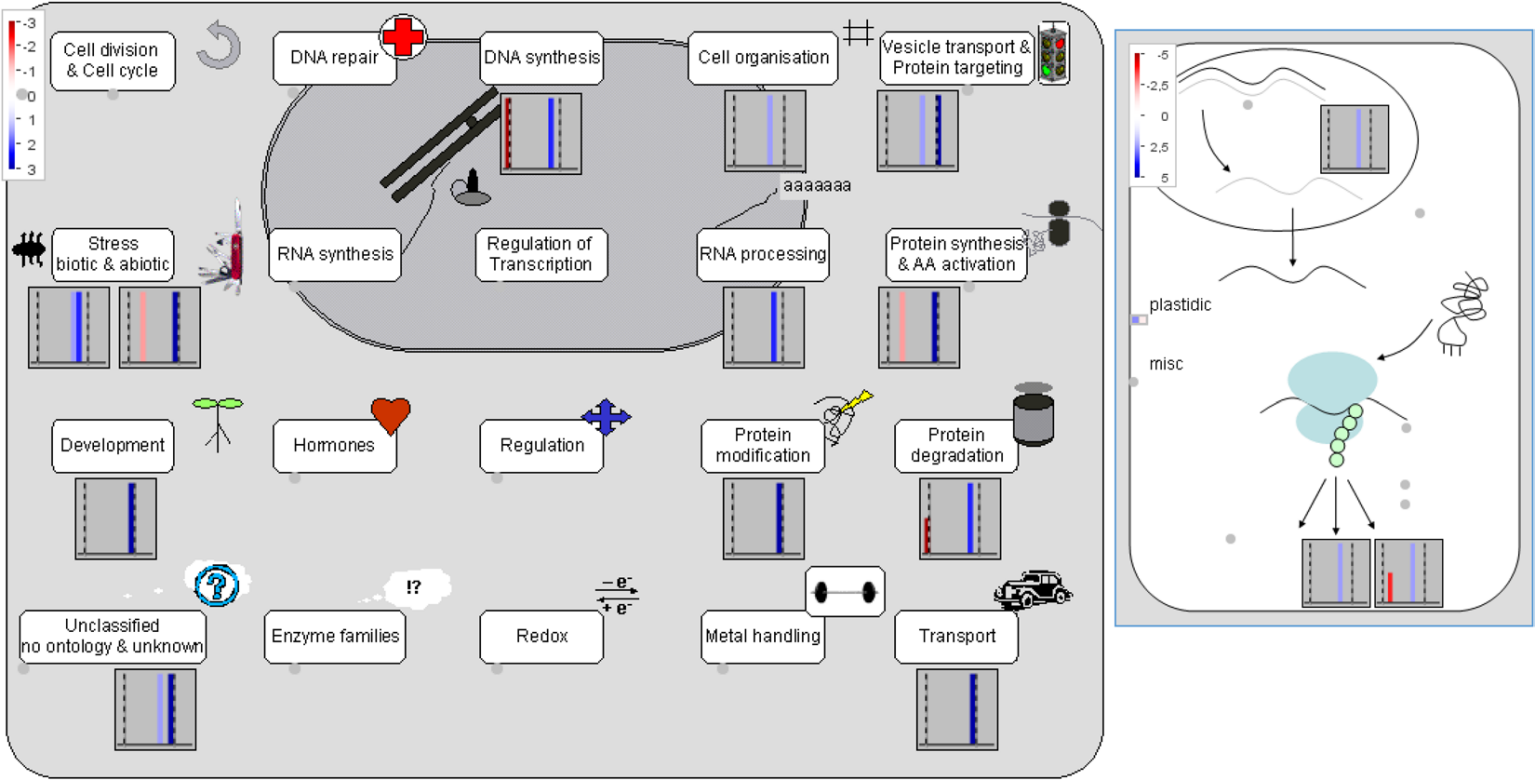
Mapman overview of cell functions in response of maize to treatment with HA (**A**) and the response of the cytoplasmic ribosomes in the category of RNA-protein synthesis that was affected by HA (**B**). The red and blue colors indicate down-regulation and up-regulation, respectively.

All data were statistically analyzed using the PatternLab TFold module. The complete list of peptides with modified expression and the corresponding list of up- and down-regulated proteins are provided in Supplementary Table 2. According to the statistical analysis, the label-free method was able to identify 43 differentially expressed proteins, significant at the p ≤ 0.05 level (Table 1); 34 proteins were up-regulated and 9 were down-regulated in plants cultivated with HA. Figure 4 shows the Volcano plot of differentially expressed proteins generated by the PatternLab program. The identified proteins are displayed on a log2 scale (fold change) on the ordinate (y-axis) and a log10 scale of the calculated probability (p-value) on the abscissa (x-axis). Blue dots represent the differentially expressed proteins that satisfied both the fold-change cut-off value and statistical criteria (p ≤ 0.05, F-stringency of 0.07 and Q-value of 0.05). These proteins are involved in different biological processes, such as glycolysis, the formation of structural constituents of the cytoskeleton, translation elongation factor activity, response to oxidative stress and endoplasmic reticulum to Golgi vesicle-mediated transport. The expression of differentially expressed proteins was visualized using Mapman software^14^ (Fig. 5). When mapped to cell function, 24 proteins were involved in protein modification, RNA processing, vesicle transport and protein targeting, cell organization, RNA-protein synthesis, transport and development.

**Table 1:** List of differentially expressed proteins in maize plants cultivated with humic acids.

Uniprot ID	Description	Fold Change	p-Value
C4J9M7	2-cys peroxiredoxin BAS1	4.2	1.2E-03
K7U392	Putative VHS/GAT domain containing family protein	3.5	0.02
K7VKG2	Glutamine synthetase3 isoform 1	3.0	1.0E-05
Q6VWJ0	Caffeoyl-CoA 3-O-methyltransferase 1	2.8	1.0E-05
B6SGM5	Putative oxidoreductase aldo/keto reductase family	2.8	0.02
C0P9T7	Uncharacterized protein	2.8	0.03
B6TVN0	40 S ribosomal protein S16	2.7	0.03
C0P861	Protein transport protein Sec. 24-like CEF	2.7	0.01
B6TTA1	Cellulase containing protein	2.6	0.03
K7UJ27	Elongation factor 1-alpha	2.5	1.0E-05
B4FKL1	Putative snRK/SAPK family protein kinase	2.5	1.0E-05
D1ME28	Uncharacterized protein	2.5	1.0E-05
K7WEL8	Uncharacterized protein	2.4	3.4E-03
K7VX77	Hexokinase	2.4	1.0E-05
B6T9A0	Histone H2B	2.3	0.01
K7V792	Uncharacterized protein	2.3	3.7E-03
B4FZ29	Argininosuccinate synthase	2.2	0.02
B6T8I3	Disease resistance response protein 206	2.1	0.04
B4FH88	C-1-tetrahydrofolate synthase. cytoplasmic	2.1	1.0E-05
B6TLS0	Ubiquitin carboxyl-terminal hydrolase isozyme L3	2.1	1.0E-05
K7UY19	Fructose-bisphosphate aldolase	2.0	1.0E-05
B6TDT1	26 S protease regulatory subunit 6B	1.9	0.03
B4FWD0	Minor allergen Alt a 7	1.9	0.01
C0P664	Tubulin beta chain	1.9	1.0E-05
B4FS87	Glyceraldehyde-3-phosphate dehydrogenase	1.9	0.02
B4FRF0	Glutathione peroxidase	1.8	0.01
C0P4Q3	Putative heat shock protein 90 family protein	1.7	1.3E-03
K7UBP7	Uncharacterized protein	1.6	1.0E-05
O50018	Elongation factor 1-alpha	1.3	1.0E-05
B4G0V4	Coatomer subunit delta	1.2	1.0E-05
B8A0C4	Uncharacterized protein	1.2	1.0E-05
C0P6Q4	Transcribed sequence 1087 protein	1.2	1.0E-05
K7V7B1	Transketolase isoform 1	1.2	1.0E-05
B6TBM1	Alpha-soluble NSF attachment protein	1.1	1.0E-05
B4FJ27	40 S ribosomal protein S24	−1.1	1.0E-05
B4FAV5	Germin-like protein 1	−1.2	1.0E-05
B4FCP0	Profilin	−1.5	4.8E-03
B4FWP0	Fructose-bisphosphate aldolase	−1.6	1.0E-05
K7UX63	Phenylalanine ammonia-lyase	−1.8	0.03
B4FTC8	3-oxoacyl-reductase	−1.8	0.02
B4G0U5	Glycoside hydrolase family 28	−2.2	0.02
C0HIB6	Histone H2A	−3.2	1.0E-05
C4J0N7	Cysteine proteinase Mir2	−3.6	3.5E-03

## Discussion

In the present study, the application of HA promoted increases in the fresh weight and root surface area of maize plants (Fig. 1). This overall increase may be associated with the stimulation of PM H^+^-ATPase^7^ that plays an important role in ion uptake and the formation of an electrochemical gradient essential in the mechanism of growth under acidic conditions. The transport of H^+^ ions occurs as the hormone auxin triggers the loosening of the cell wall, resulting in plant growth^8^. A putative HA hormone-like activity has been hypothesized to affect plant roots grown in media with a low nutrient concentration^15^. Zandonadi *et al*. evaluated the effects of indole-3-acetic acid (IAA) and HA on maize root development and observed that at low concentrations (10^−10^ and 10^−15^ M), HA and IAA stimulated the lateral roots and activated the PM and vacuolar H^+^-ATPases and H^+^-pyrophosphatase.

The categories presented in the Figs 2 and 3 suggest that HA significantly influences primary and secondary metabolism, which is reflected in biomass increase. Our results show that, in plants treated with HA, there was a greater expression of proteins involved in the glycolytic pathway. This pathway enables the biochemical adaptation of species to environmental stresses, such as anoxia, nutrient limitation and osmotic stress^16^. After treatment with HA, GAPDH was up-regulated (Table 1; Fig. 5). Moreover, HAs are active in carbon and nitrogen metabolism by increasing the activities of enzymes in glycolysis, the Krebs cycle and nitrogen assimilation. In maize seedlings treated with HA, the enzymes involved in glycolysis increased. Therefore, HA positively affects the physiological processes of the plant, primarily by increasing the enzymes in the glycolytic pathway. However, the concentrations of HA, age and plant species determine whether the overall effect is inhibitory or stimulatory^17^. Canellas *et al*. observed that while HS decrease the amount of carbohydrates, such as glucose and fructose, in the leaves of maize seedlings, they increase the starch content. They also observed the expression of enzymes that function in plant metabolism and in mobilizing storage proteins in the early stages of germination, thus aiding the development of seedlings^18^. Mass spectrometry revealed that glutamine synthetase (GS), the enzyme involved in N assimilation, was up-regulated in plants treated with HA (Table 1; Fig. 5). Nitrate reductase (NR) and glutamine synthetase (GS) are crucial metabolic enzymes involved in the conversion of simple forms of inorganic N^19^. GS plays an important role in fixing ammonium to form the amino acid glutamine^20^. Other studies have also observed that application of HA to maize seedlings increases the activity of GS^21^. We also observed that the functional category “cellular nitrogen compound metabolic process” (Fig. 2) was represented by more proteins than control. Therefore, HA contributes to increased activity of enzymes involved in N metabolism, which is important for plant development.

Interestingly, several antioxidant enzymes were differentially detected, among them 2-Cys peroxiredoxin BAS1, a putative heat shock protein 90 family protein, glutathione peroxidase and histone H2B were overexpressed in the plant roots grown with HA. Peroxides, such as H_2_O_2_ are produced during normal aerobic cellular processes and can be converted to free radicals that cause cellular damage^22^. The accelerated production of reactive oxygen species (ROS) indicates a plant in physiological stress. Peroxiredoxins are efficient antioxidants protecting against biotic and abiotic stresses by decomposing hydrogen peroxide. In transgenic *Arabidopsis* with decreased levels of 2-Cys Prx, photosynthesis was impaired and the plant developmental stages showed an increase in oxidative damage^23^. Glutathione peroxidase (GPX) is an antioxidant enzyme able to scavenge a single type of ROS^24^. Several roles for glutathione have been suggested, including protecting growth and development from the effects of heavy metals, enabling the tolerance to adverse environmental conditions that promote oxidative stress^25^. On the other hand, heat shock proteins (HSPs) are found ubiquitously in plant and animal cells and are involved not just in heat shock^26^, but also in a wide variety of stresses such as salt and biotic stresses^27^. Recent studies show that Hsp90 is involved in the developmental responses of plants to stress and resistance to pathogens^28^. Therefore, the actions of HA go beyond its influence on root development, extending to a protective role in the cell. In Fig. 3, our results showed a higher number of stress response proteins in the control condition compared to plants grown in the presence of HA. This shows that the application of HA produces a positive effect on the plants by alleviating stress.

Cellular transport is one of the biological processes that was most affected in this study. We identified three proteins involved in the transport of proteins that were up-regulated: the coatomer delta subunit, the alpha-soluble NSF attachment protein and the putative VHS/GAT domain containing family protein^29^. The coatomer delta subunit moves the Golgi apparatus to the endoplasmic reticulum. It is mediated by vesicles bearing specific protein coats such as COP I (coat protein complex I), one of two multimeric complexes that form a membrane vesicle coat^30^. COP I plays an important role in protein transport in the early secretory pathway and is responsible for protein trafficking through the endomembrane system of the endoplasmic reticulum and Golgi apparatus^31^. Many substances, such as lipids and proteins, are involved in the packaging and directing of vesicles to various organelles, one of which is the soluble N-ethylmaleimide-sensitive factor (NSF). This factor, along with the α-soluble NSF attachment protein (SNAP), are key components of vesicle trafficking systems that are essential for development, growth and may be related to resistance in plants^27,32^. *A*. *thaliana* has a large number of proteins with VHS (Vps27-Hrs-STAM) and GAT (GGA and Tom) domains; these belong to the TOL (TOM1-like) proteins that are transported to the vacuole and are involved in the regulation of ubiquitinated plasma membrane proteins^33^. Exposure to HS may interfere in vesicle trafficking and transport mechanisms, indicating that these substances influence plant development and showing particularly a relationship between HS and root development. The Golgi complex plays an important role in the secretory pathway. In *Arabidopsis*, V-ATPase inhibition resulted in a blockage of protein trafficking between the Golgi complex and late endosomes^34^. V-ATPases are essential in plant development as well as for Golgi complex function^35^. The activation of vacuolar pumps (i.e. V-ATPase) is modulated by HS^36^. Therefore, these proteins could be involved in several aspects of cell function, including those involved in the endosomes and Golgi apparatus as well as other specific functions of the plant^33^. The identification of proteins by Mapman software showed that proteins of the category “vesicle transport and protein targeting” were also up-regulated (Fig. 5).

Cytoskeleton proteins, such as tubulin (tubulin beta-chain) were up-regulated in the maize roots treated with HA. Tubulin, a major component of microtubules, has an essential role in the cell cycle and cell wall construction^37^. Actin is the primary protein constituent of microfilaments and is required for the reorganization of the cytoskeleton during cell elongation in response to attacks by pathogens^38^. However, the protein profilin (PFN) was downregulated in maize roots treated with HA. PFN is an actin-binding protein that is present in various organisms, including higher plants^39^. Our results show a no-stimulatory effect of HS on this protein 11 days after planting (DAP), which suggests the need for new approaches to explore this area. In addition, Elongation factor 1-alpha (EF1α), a protein related to the dynamics of actin and microtubule filaments, was detected only in maize roots grown in the presence of HA. EF1α influences actin and microtubules in a Ca^2+^/calmodulin-dependent^40^ manner. Considering that Ca^2+^ and calmodulin are important in cell signaling in plants, this result suggests that the regulation of EF1α is a part of cytoskeletal dynamics and can act in the intercommunication of signaling pathways between the cytoskeleton and plant development^41^. Ca^2+^ plays a key role in the regulation of processes such as gene transcription, plant development and physiological processes, including signaling and adaptation to stress^42,43^. The microtubule cytoskeleton acts in the control of plant growth and salt stress has been reported to cause microtubule reorganization, resulting in plant adaptation to stress^44–46^. Lower levels of tubulin protein result in a hypersensitivity response at high salinity. Thus, cytoskeleton proteins may be important in protecting the plant against salt stress.

Protein folding-related proteins were only detected in maize roots treated with HA; these include proteins such as calnexin, calreticulin and endoplasmin-like protein. These proteins belong to the group of endoplasmic reticulum (ER)-located protein folding mediators^47^. Calreticulin, a chaperone protein involved in protein folding, acts on the regulation of intracellular Ca^2+^ homeostasis in the ER^48,49^. Calcium homeostasis, reactive oxygen species production and vesicle exocytosis are processes that influence the growth of plants^50–52^. Calnexin deficiency in plants affects their development because of incorrect protein folding, which may result in ER stress^53^. Calnexin and calreticulin are molecular chaperones that play an important role in protein folding. These proteins play a role in stress signaling and plant tolerance, forming part of the mechanisms of resistance to stress^54^.

The last category highlighted was RNA processing. In HA-treated roots, proteins involved in RNA processing were identified, such as eukaryotic initiation factor 4a and the S-adenosyl-L-methionine-dependent methyltransferase superfamily. The Mapman software analysis assigned some proteins involved in RNA processing and protein synthesis (Fig. 5). The eukaryotic translation initiation factors (eIFs) act to regulate the stages of translation initiation, playing important roles in the growth, development and reproduction processes of plants^55^. The S-adenosyl-L-methionine-dependent methyltransferase superfamily acts in post-translational modification processes, where this enzyme catalyzes the methylation of proteins by S-adenosyl-L-methionine-dependent methyltransferases. Protein modification plays a key role in many cellular functions, such as plant growth and development^56^. Some differentially expressed proteins, evaluated through Mapman software, are involved in RNA processing. As shown in Fig. 3, the categories of “translation” and “biosynthetic processes” represented higher numbers of proteins. Thus, our results show that HA can modify protein synthesis pathways, influencing root development. Plants modulate gene expression to adapt to the challenges under biotic and abiotic stresses. These changes include molecular mechanisms of RNA processing^57^. Initiation factors, such as eIF4A, may be related to the regulation of translation in response to abiotic stress in plants, conferring salt tolerance in *Arabidopsis* plants^58^.

## Conclusions

The effects on root architecture, such as induction of lateral root growth and biomass increase, were accompanied by changes in proteins involved in energy metabolism, protein folding, cytoskeleton organization, RNA processing, stress response, N assimilation, transport of proteins and vesicle transport. Among these proteins, we have highlighted the ones that are involved with vesicle transport, such as the putative VHS/GAT domain-containing family of proteins with antioxidant responses, the 2-Cys peroxiredoxin BAS1 involved in the assimilation of N, and glutamine synthetase 3 isoform 1. The modifications influenced by the effect of HA on the roots of maize plants represent tools of great importance for agriculture.

## Materials and Methods

### Humic acids

Humic acids (HA) were extracted from 10 g of vermicompost, prepared using cattle manure and *Eisenia foetida* with 100 mL of a 0.1 M NaOH solution under an N_2_ atmosphere. This procedure was repeated several times until the supernatant became colorless. Extracts were then combined and centrifuged at 5000 × g for 15 min. The supernatant was then acidified with 6 M HCl to pH 2.0 and kept at 4 °C for 12 h. The precipitated HA was separated by centrifugation from the soluble fulvic acid that remained in the supernatant. The HA was purified by treating it three times with 10 mL of a dilute HF (0.3 M) + HCl (0.1 M) solution. After centrifuging at 4000 × g for 15 min, the sample was washed repeatedly with water, dialyzed against deionized water using a 1 kDa cut off membrane, and lyophilized to maintain stability until use. The HA elemental composition was determined using a CHNS/O PerkinElmer auto analyzer (PerkinElmer 2400 Series II, Norwalk, CT, USA). The O content was calculated from the difference (i.e., O% = 100 – C% – H% – N%) on an ash-free basis and the elemental composition was: C = 47%, H = 5.1%; N = 3.1%; O = 43%.

### Plant growth and addition of humic acids

Maize seeds (*Zea mays* L. DKB 789) were surface-sterilized by soaking in 0.5% (w/v) NaCl for 30 min, followed by rinsing and then soaking in sterilized distilled water for 6 h. The seeds were transferred to glass jars containing 30 mL of 0.6% aqueous agar (Sigma-Aldrich, St. Louis, MO, USA) and were maintained for 3 days in a growth chamber at 27 °C in the dark for germination. Four-day-old maize seedlings with roots approximately 4 cm long were transferred to 1 L plastic vessels containing 2 mM CaCl_2_ for 7 days. Minimal medium (CaCl_2_) was used to avoid any influence of nutrients that could act synergistically with HA and stimulate root growth and metabolism of the seedlings^6^. The solutions were adjusted to a pH of 6.0 and continuously aerated by a low flux pump (0.014 MPa) used in aquarium systems. Two growth conditions were compared in the experiment: minimal medium with HA at 50 mg CL^−1^ (3.5 mM C) and the control grown without HA. Previous experiments showed that seedlings treated for 3 days with 50 mg CL^−1^ HA exhibited a higher number of lateral roots compared with the control plants and seedlings treated for 7 days with 40 mg CL^−1^ exhibited a proliferation of the mitotic sites in this zone of roots^6^. Fifteen seedlings were placed on a plate perforated with 15-mm-diameter holes. The seedlings were grown for 7 days under a 12/12 light/dark regime at 27 °C, 60% relative humidity and a light intensity of 45 µmol m^−2^ s^−1^ and collected 11 days after planting (DAP). The seeds were germinated for 4 days and plants were cultivated for 7 days.

### Assessment of growth parameters

At 11 DAP, maize seedlings were collected to measure fresh weight. Plants separated for proteomic analysis were stored at −80 °C until protein extraction. Fresh weight data was analyzed with parametric t-tests. The roots were separated, placed on glass plates, and scanned for imaging. SAFIRA® software^59^ was used to analyze images and calculate the root’s area total surface. Data was analyzed using GraphPad Prism (Version 6.0), and significant differences were tested using the t-test.

### Protein extraction

Proteins were extracted using a previously described protocol^60^ with some modifications as follows. Protein precipitation was conducted using 500 mg of root tissue pooled from five roots obtained from the control and HA-treated plants harvested at 11 DAP, resulting in three protein extracts used for each treatment. The experiments were reproduced in biological triplicates for each treatment. Frozen roots were ground to a fine powder in liquid nitrogen using a pestle and mortar. The proteins from each sample were extracted using 4 mL of extraction buffer (0.5 M Tris-HCl, pH 8; 0.7 M sucrose; 100 mM EDTA; 1% (w/v) CHAPS; 14 mM DTT; 20 mg/mL of Roche protease inhibitor) and shaken with a vortex mixer to homogenize. After centrifuging at 16,000 × g for 20 min at 4 °C, the supernatant was transferred to a new tube and precipitation buffer (12.5% (w/v) TCA and 0.125% (w/v) DTT in pure acetone) was added. Pellets were incubated at −20 °C for 1 h and centrifuged at 16,000 × g for 20 min at 4 °C, then washed three times with pure methanol, twice with pure acetone, and once with acetone containing 0.1% (w/v) DTT. This was followed by centrifuging at 10,000 × g for 30 min at 4 °C. The dried protein pellets were then suspended in 25 mM ammonium bicarbonate and 0.5 M urea. After sonicating for 30 min in ice and centrifuging at 10,000 × g for 30 min at 15 °C, the proteins in the supernatant were quantified using the Bradford assay^61^.

### Enzymatic digestion

An aliquot of each sample (1 mg/mL) was first treated with 10 mM DTT for 30 min and then with 50 mM iodoacetamide for 30 min in the dark. Trypsin (Promega, Madison, WI, USA) (1:50, w/w) was added to the sample for overnight digestion at 37 °C. All samples were vacuum-dried and reconstituted using 0.1% formic acid. Three biological replicates were produced for each of the control and HA-treated plants.

### Nanoflow liquid chromatography coupled with LTQ Orbitrap Velos

An aliquot (4.5 µL) of each particular sample was loaded onto an LTQ Orbitrap Velos mass spectrometer (Thermo Fisher Scientific, Waltham, MA), which was connected to a Nano flow LC (nLC-MS/MS) with an EASY-nLC system (Proxeon Biosystems, West Palm Beach, FL, USA) through a Proxeon Nanoelectrospray ion source. Peptides were separated by a 2–90% acetonitrile (ACN) gradient in 0.1% formic acid using an analytical PicoFrit® column (20 cm × ID 75 mm, 5 mm particle size, New Objective, Woburn, MA), at a flow rate of 300 nL/min over 45 min. The Nano electrospray voltage was set to 1.7 kV and the source temperature was 275 °C. All operational procedures for the LTQ Orbitrap Velos were input in the data-dependent acquisition mode. The full scan MS spectrum (m/z 300–1,600) was obtained from the Orbitrap analyzer after accumulation to a target value of 1e6. Resolution in the Orbitrap was set to r = 60,000 and the 20 most intense peptide ions with charge states ≥2 were sequentially isolated to a target value of 5,000 and fragmented in the linear ion trap by low-energy collision induced dissociation (CID), with a normalized collision energy of 35%. The signal threshold for triggering an MS/MS event was set to 500 counts. Dynamic exclusion was enabled with an exclusion size list of 500, an exclusion duration of 60 s, and a repeat count of 1. An activation q of 0.25 and activation time of 10 ms was used.

### Database searching and statistical analysis

Initially, the RAW files were viewed in Xcalibur v.2.2 (Thermo Scientific) and a subsequent data search was conducted of the *Zea mays* database downloaded from Uniprot in September 2015, using PatternLab (v.4.0.0.5) for the proteomics computational environment^62^ and the Comet algorithm (v.20144.011)^63^. Parameters used were as follows: fully tryptic peptides; up to two missed cleavages; carbamidomethylation as fixed modification; oxidation of methionine as a variable modification; and a peptide precursor tolerance of 40 ppm. The Search Engine Processor^64^ was used for post-processing of the peptide spectrum matches to achieve a protein list with less than 1% false discovery range (FDR). Proteins identified in at least two replicate samples were considered for further analysis. The PatternLab TFold module was used to identify differentially expressed proteins in the two treatments^63^. The TFold module uses a p-value-dependent fold-change cutoff to maximize the number of identifications that satisfy a theoretical FDR estimator (in this case, the Benjamini-Hochberg). Fold-changes were calculated as the average spectral counts obtained in the reference control roots (CR) divided by those obtained in humic acid roots (HAR).

### Bioinformatics analysis of identified proteins

Gene Ontology (GO) annotations for Molecular Function categories were obtained using the AgBase tools and database (http://agbase.msstate.edu/index.html). GOanna was used to determine the GO annotation and orthologs for all proteins using the Uniprot Database as a reference. Plant GOSlim was used to summarize the Molecular Function category of each of the identified proteins. Enrichment analysis of the maize root proteins was executed by the Singular Enrichment Analysis tool from the AgriGO website (http://bioinfo.cau.edu.cn/agriGO/)^13^. Differentially expressed proteins related to cell functions were visualized using the MapMan software (http://mapman.gabipd.org)^14^. The proteomics datasets produced in this study are available in the PRIDE database using the following accessions: PXD014407.

## Supplementary information

List of proteins identified

List of proteins with modified expression
